# circSYPL1 Promotes the Proliferation and Metastasis of Hepatocellular Carcinoma via the Upregulation of EZH2 Expression by Competing with hsa-miR-506-3p

**DOI:** 10.1155/2022/2659563

**Published:** 2022-03-19

**Authors:** Da Lei, Tao Wang

**Affiliations:** Department of Hepatopancreatobiliary and Spleen Surgery, Baoji Central Hospital, Baoji 721008, Shannxi Province, China

## Abstract

**Objective:**

Circular RNAs (circRNAs) and microRNAs are crucial for progressing of hepatocellular carcinoma (HCC). Nonetheless, the function or mechanisms of a newly discovered circRNA, circSYPL1, as well as miR-506-3p, in the progression of HCC are mostly unexplained. The purpose of this research was to determine the mechanisms by which circSYPL1 and miR-506-3p regulate the malignant features of HCC.

**Methods:**

The expression level of circSYPL1 was indeed detected using real-time PCR in HCC cell lines, primary as well as metastatic cancers. To assess the functionality of circSYPL1 upregulation and knockdown, we used proliferation and apoptosis, in addition to migration assays, as well as tumor xenograft and lung metastasis assays. The mechanisms of competing endogenous RNAs with circSYPL1/miR-506-3p/EZH2 were investigated using luciferase as well as RNA pull-down experiments. Lastly, cell proliferation and migration, in addition to tumor xenograft tests, were used to validate the biological significance of the circSYPL1/miR-506-3p/EZH2 signaling axis through overexpression or otherwise silencing.

**Results:**

circSYPL1 expression was significantly upregulated in HCC cell lines, in addition to primary and metastatic tumors of patients with HCC. Additionally, it may promote HCC initiation, development as well as progression. By knocking down circSYPL1 siRNA, we were able to drastically decrease the aggressiveness of HCC cells. circSYPL1 sponged miR-506-3p to boost EZH2 expression levels, as indicated by luciferase and RNA pull-down assays. Furthermore, circSYPL1 overexpression could upregulate EZH2 expression, while miR-506-3p mimics or EZH2 shRNAs could reverse the circSYPL1-induced malignancy of HCC cells.

**Conclusion:**

On a mechanistic level, circSYPL1 can interact with miR-506-3p in a competitive manner to upregulate EZH2, hence increasing the aggressiveness of tumors.

## 1. Introduction

Hepatocellular carcinoma (HCC) is a primary liver cancer that develops more frequently in individuals who have pre-existing chronic liver disease or cirrhosis [1]. It represents the fourth leading reason of cancer death and is currently ranked sixth on the basis of global incidence [1]. Its onset is strongly related to uncontrolled cell proliferation and unregulated metastasis [2]. More than one million people will die worldwide from liver malignancies by 2030, as per the World Health Organization (WHO) [1]. HCC is the second commonest fatal malignancy in the United States, with an 18% five–year survival rate [3]. The pathophysiology of HCC is not completely established at the moment. Consequently, it is critical to understand the molecular pathways underlying HCC pathogenesis in order to develop promising therapeutics.

Circular RNAs (circRNAs) are a subgroup of endogenous noncoding RNAs They have the capacity to impact gene expression levels before and after the completion of transcription [4]. They have always been considered promising diagnostic and prognostic markers as they are implicated in the pathogenesis of several malignancies, particularly HCC [5]. For example, circZKSCAN1 [6] and cSMARCA5 [7, 8] are deregulated in tumor tissues and plasma of patients with HCC. Certain circRNAs such as circASAP1 [4], circSMAD2 [9], circMTO1 [10], circSETD3 [9], circHIPK3 [11], and circFBLIM1 [12], either as oncogenic or suppressive genes, can affect HCC aggressiveness as well as programmed cell death (apoptosis). These circRNAs may certainly assist in finding new diagnostic and prognostic markers as well as promising therapeutic strategies for HCC, if we fully comprehend the molecular mechanisms as well as signaling pathways behind them. MicroRNAs (miRNAs) are short noncoding RNA biomolecules which target the 3′-UTR of genes to inhibit their expression [13, 14]. MiRNAs have been shown to perform a critical function in a variety of liver cancers, particularly HCC [15, 16].

Small noncoding RNA molecules, known as microRNAs, specifically target the 3′-UTR for regulating gene expression [13, 17]. miRNAs have a significant function in a number of liver cancers, particularly HCC. Using miRNA-506-3p, He and Wang [14] discovered that osteosarcoma (OS) cell proliferation as well as metastasis were inhibited. miR-506 also inhibits neuroblastoma metastasis mostly by targeting ROCK1 [15], while miR-506 decreased expression promotes pancreatic tumor progression [16]. Using miR-506 to limit the aggressiveness of nasopharyngeal cancer, researchers were able to inactivate the Wnt/beta-catenin signaling pathway by downregulating LHX2 [18]. miR-506-3p could also reduce autophagy and stimulate MET in OS cells by specifically targeting the SPHK1 gene, according to previous research [19]. GALNT4 is a direct target of the microRNA miR-506-3p, which is known to slow the development of prostate cancer [20]. miR-506-3p could also modulate the SIRT1/AKT/FOXO3a signaling pathway in ovarian cancer cells, inhibiting proliferation and promoting apoptosis [21]. Additionally, miR-506 can inhibit hepatoma cellular proliferation by specifically targeting YAP mRNA [22]. Such findings imply that miR-506 performs a critical function in cancer progression.

We identified a new circRNA that modulates HCC pathogenesis through the mechanism of competing endogenous RNAs (ceRNAs). We originally discovered that the upregulation of circRNA circSYPL1 in HCC cell lines and primary and metastatic tumors of HCC patients facilitates tumor xenograft progression and aggressiveness. Furthermore, it can competitively sponge with miR-506-3p to upregulate the downstream molecule EZH2 to enhance the malignancy of tumors. miR-506-3p overexpression or EZH2 knockdown could reverse liver cancer progression induced by circSYPL1. Thus, the circSYPL1/miR-506-3p/EZH2 axis may be a promising target molecule for treating and diagnosing HCC.

## 2. Materials and Methods

### 2.1. Cell Culture

Before incubation under 37°C and 5% CO_2_, cancer cell lines (7402, 7721, HepG2, and PLC-PRF-5), in addition to normal liver cells (LO2), underwent cultivation in DMEM with the supplementation of 10% FBS as well as 1% penicillin/streptomycin.

### 2.2. Tissue Samples

During surgeries at Baoji Central Hospital, eleven pairs of renal malignancy and nearby normal tissue specimens were collected. This hospital's Institutional Review Board (IRB) has authorized all trials, and the patients have given their written consent. The samples were preserved at −80°C after being frozen in liquid nitrogen.

### 2.3. Cell Transfection

RiboBio supplied the circSYPL1 siRNA, miR-506-3p mimics, EZH2 siRNA, and negative control (Guangzhou, China). Our lab developed circSYPL1 or EZH2 WT and MT plasmids and their blank plasmids. Seeding HepG2 and PLC-PRF-5 cell lines into 12-well plates for 12 hours prior to transfection was carried out. Lipofectamine 2000 Invitrogen was used to transfect recombinant plasmid, circSYPL1 siRNA, miR-506-3p mimics, as well as EZH2 siRNA for transient transfection. Following 24 hours, specimens were taken to assess the upregulation or downregulation of the target gene.

### 2.4. RT-PCR

TRIzol was used to homogenize tissue specimens. The manufacturer's instructions were followed for the extraction and reverse transcription of total RNA. In order to perform PCR on the cDNA fragment that encodes the target genes, the sense and antisense primer sequences for circSYPL1, miR-506-3p, EZH2, as well as *β*-actin were utilized [14].

### 2.5. BrdU Assay

In 96-well plates, 3000 HepG2 and PLC-PRF-5 cells were seeded per well. The BrdU kit was used to evaluate for cellular proliferation (Beyotime, Shanghai, China). Finally, the fluorescent microscope was used to examine the cells in three chosen random visual fields. The cellular proliferation rate is computed using this formula. The fluorescence intensity was calculated using the software in the Leica DMI3000B microscope.

### 2.6. TUNEL Assay

Assessing the apoptotic activity was achieved using this assay. Then, DAPI was utilized for staining the nuclei. Finally, the apoptotic cells were captured through fluorescence microscopy. The experiment had three replicates.

### 2.7. Luciferase Assays

Components of miR-506-3p binding site-containing vectors were used to introduce complementary as well as mutant sequences of circlesYPL1 and circlesEZH2 into the dual-luciferase reporter genes. Cotransfection of HEK 293T cells with miR-506-3p or control mimics and a firefly luciferase reporter vector encoding WT or mutant circSYPL1 and EZH2 sequences was performed using the Lipofectamine 2000 reagent. Measurement of luciferase activity 24 hours after transfection was performed using the Dual-Luciferase® System.

### 2.8. RNA Pull-Down Assay

circSYPL1 and miR-506-3p were labelled with biotin to create biotin-miR-506-3p and biotin-circSYPL1, respectively. They were therefore incubated with cell lysates as well as magnetic beads coated with streptavidin were administered (Life Technologies). The amounts of circSYPL1, EZH2, as well as miR-506-3p were quantified by quantitative PCR (qPCR).

### 2.9. Cell Migration Assays

The circSYPL1 recombinant protein and negative control plasmid, circSYPL1 coupled with miR-506-3p mimics, or circSYPL1 together with sh-EZH2 were transfected in 12-well plates (10,000 cells/well) for 24 hours in liver cancer cell lines. Subsequently, 5000 cells for each well were seeded in the inserts of Transwells with 8-micron-pores that had been pre-equilibrated (Corning, USA). Inserts were subsequently rinsed, completely fixed in 2% paraformaldehyde for 10 minutes, and stained with crystal violet (CV) just after 24 hours of incubation (Beyotime, Jiangsu, China). A microscope was used to quantify cells that had migrated (ECLIPSE Ti, Nikon, Japan).

### 2.10. Tumor Xenograft

Lenti-NC and Lenti-circSYPL1-treated cells were subsequently administered as a subcutaneous (SC) injection into the right leg of nude mice. In the tumor-bearing mouse models, researchers used the PLC-PRF-5 cell line. For the duration of the study, the tumor volumes were determined. Tumor's size calculated as follows: tumor volume (V/mm^3^) = length × width^2^/2 (longest diameter (a) and shortest diameter (b)). A surgical procedure was therefore performed to eradicate the tumor xenografts, and the tumor mass was weighed. This research study was authorized by the Baoji Central Hospital IRB.

### 2.11. Statistical Analysis

Every test was replicated three times. The collected data were analyzed by the GraphPad Prism 8.0 program, with a two-tailed, unpaired Student's *t*-test as well as a one-way ANOVA as the primary statistical methods. *P* < 0.05 was considered significant.

## 3. Results

### 3.1. circSYPL1 Levels

**Figure 1:**
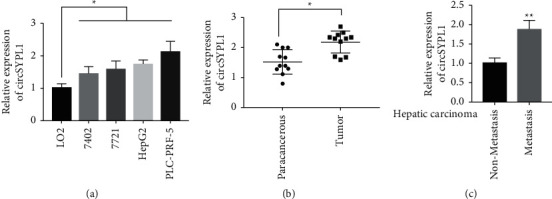
circSYPL1 expression levels. (a) qPCR quantify the levels of circSYPL1 in normal liver cells (LO2) versus liver cancer cells. (b) qPCR analysis demonstrating the expression of circSYPL1 in liver cancer tumors and surrounding tissues. (c) qPCR analysis demonstrating the expression of circSYPL1 in liver cancer tissue from patients with and without metastases.

To examine the expression of circSYPL1 in participants with liver malignancy and cell lines, we used qPCR to evaluate the expression of circSYPL1 in primary and metastatic liver cancerous tissue as well as cell lines. The real RT-PCR analysis revealed upregulated circSYPL1 in liver cancer cells when compared to healthy liver cells (LO2) (Figure 1(a)). Subsequently, using qPCR, we evaluated the expression of circSYPL1 in a total of 20 matched liver cancers and neighboring healthy tissues. The outcomes showed a relative upregulation of circSYPL1 in liver cancer samples in comparison to adjacent healthy tissue specimens (Figure 1(b)). Additionally, we examined the expression of circSYPL1 in metastasized and nonmetastasized tumor tissues and discovered that it was upregulated in the former than the latter (Figure 1(c)).

### 3.2. Silencing circSYPL1 Suppresses the Malignant Features

**Figure 2:**
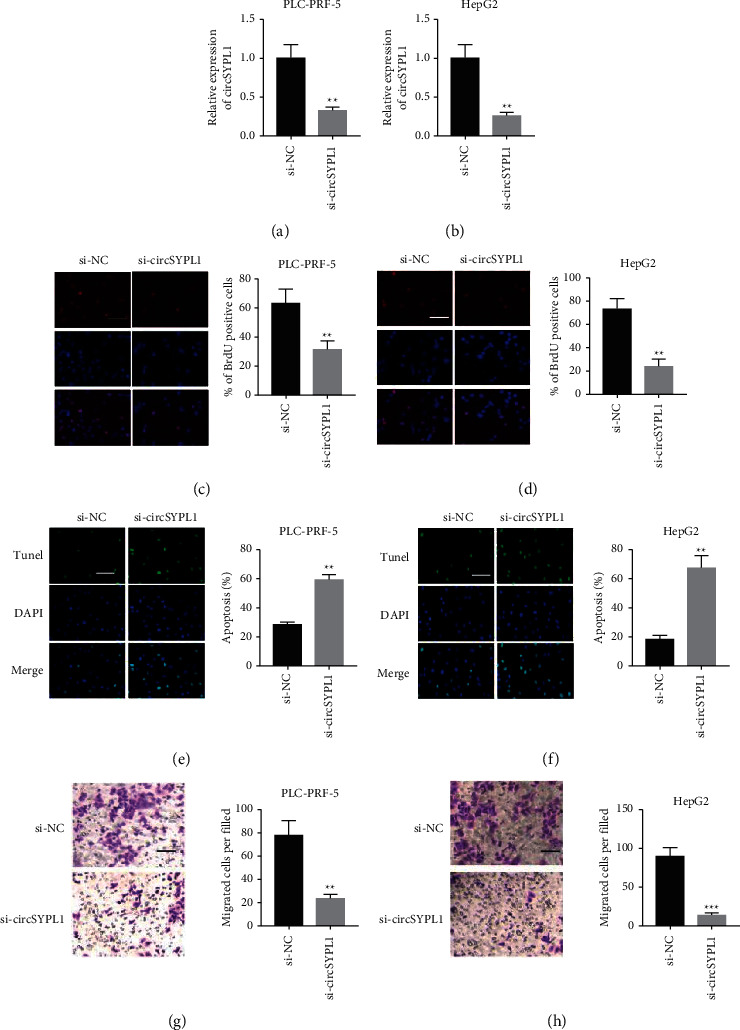
In vitro, silencing circSYPL1 decreases proliferation and metastasis of HCC but enhances apoptosis. (a) qPCR showing the level of circSYPL1 in PLC-PRF-5 cells treated with circSYPL1 or negative control siRNAs. (b) qPCR showing the expression level of circSYPL1 in HepG2 cells treated with circSYPL1 or negative control siRNAs. (c) Cell proliferation assay of PLC-PRF-5 cells treated with circSYPL1 or negative control siRNAs by the BrdU test. (d) Cell proliferation assay of HepG2 cells treated with circSYPL1 or negative control siRNAs by the BrdU test. (e) TUNEL assay detected the apoptosis of cisplatin-induced PLC-PRF-5 cells treated with circSYPL1 or negative control siRNAs. (f) TUNEL assay detected the apoptosis of cisplatin-induced HepG2 cells treated with circSYPL1 or negative control siRNAs. (g) Representative CV staining images (left) as well as statistical analyses (right) of a transwell invasion assay using PLC-PRF-5 cells treated with circSYPL1 or a negative control siRNA. (h) Representative CV (left) in addition to statistical analyses (right) of a transwell invasion assay using HepG2 cells treated with circSYPL1 or a negative control siRNA.

To determine the function of circSYPL1 in liver cancer progression, treating of cancer cell lines with circSYPL1 siRNA was conducted. The data demonstrated that circSYPL1 siRNA significantly downregulated circSYPL1 (Figures 2(a) and 2(b)) and subsequently reduced cell survival (Figures 2(c) and 2(d)). TUNEL assay was used to determine apoptotic activity. The si-circSYPL1 group was found to have a greater proportion of apoptotic cells than the siRNA control group (Figures 2(e) and 2(f)). Additionally, we examined the influence of circSYPL1 utilizing a transwell assay and discovered that the group treated with circSYPL1 siRNA had a smaller percentage of PLC-PRF-5 or HepG2 cells than the group treated with no siRNA (Figures 2(g) and 2(h)). These results demonstrate that circSYPL1 promotes the aggressiveness of liver malignancies while inhibiting apoptosis.

### 3.3. Silencing circSYPL1 Inhibits the Aggressiveness of Liver Tumor Xenograft

**Figure 3:**
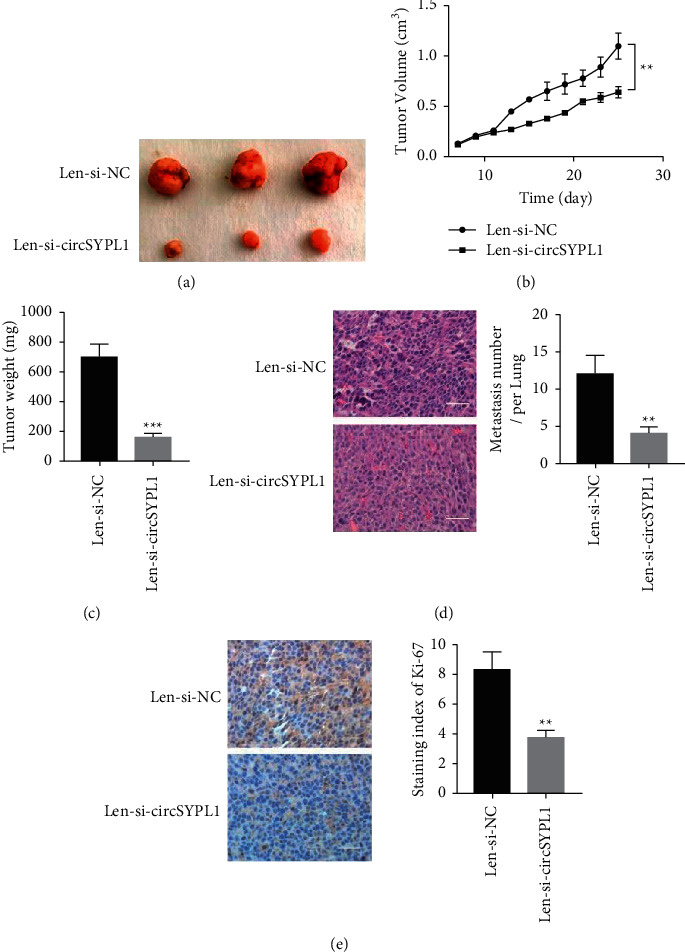
Knockdown of circSYPL1 inhibits the growth and metastasis of tumor xenografts. (a) Photographs of PLC-PRF-5 isolated from animal models treated with circSYPL1 siRNA or a negative control siRNA. (b) Growth rates of circSYPL1 or negative control siRNA-treated PLC-PRF-5. (c) Tumor mass of PLC-PRF-5 in groups treated with circSYPL1 or negative control siRNAs. (d) HE is staining analysis of lung metastasis in groups treated with circSYPL1 or negative control siRNAs. (e) Immunohistochemistry staining analysis of tumor proliferation (Ki-67) in groups treated with circSYPL1 or negative control siRNAs.

To examine the function of circSYPL1 in tumor formation in vivo, we administered 1 × 106 PLC-PRF-5 cells with a lentivirus overexpressing circSYPL1 in two months-old nude mice. The PLC-PRF-5 xenograft injected with lenti-circSYPL1 was significantly smaller than the control lenti-negative xenograft (Figure 3(a)). Additionally, we assessed tumor volume from day 7 to day 25 following injection and discovered that the lenti-circSYPL1 group had a much reduced tumor volume than the lenti-negative control group (Figure 3(b)). Additionally, we discovered that the lenti-circSYPL1 group's tumor mass was noticeably lighter than that of the lenti-negative control group (Figure 3(c)). Additionally, H&E staining of lung tissues revealed that the lenti-circSYPL1 group's metastatic area was significantly less than that of the lenti-negative control group (Figure 3(d)). Ki-67 immunohistochemistry staining analysis demonstrated that the lenti-circSYPL1 group had significantly decreased tumor cell proliferation compared with the negative control siRNA-treated groups (Figure 3(e)). These outcomes revealed that circSYPL1 could promote the aggressiveness of PLC-PRF-5 tumor xenografts.

### 3.4. circSYPL1 Interacts with miR-506-3p

**Figure 4:**
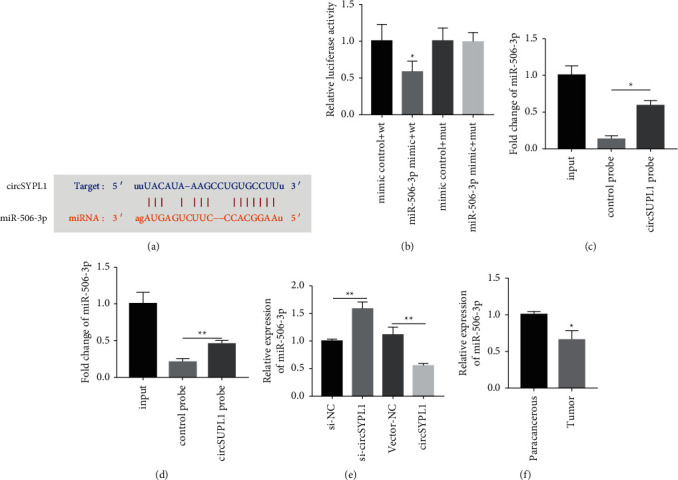
circSYPL1, miR-506-3p interactions. (a) miR-506-3p (orange) and circSYPL1 interacting sequences (blue). (b) Luciferase assays to demonstrate that circSYPL1 is directly targeted to miR-506-3p. (c) RNA pull-down assay to determine the interactions between circSYPL1 and miR-506-3p using a biotinylated circSYPL1 probe. (d) RNA pull-down assay to determine whether the miR-506-3p biotin probe interacts with circSYPL1. (e) qPCR demonstrating miR-506-3p expression in cells after treatment with circSYPL1 siRNA or its recombinant overexpression plasmid. (f) qPCR analysis showing the levels of miR-506-3p in HCC and surrounding tissues.

To determine if circSYPL1 functions as a ceRNA by sponging miRNA, we conducted alignment experiments and discovered that miR-506-3p may have the ability to target circSYPL1 (Figure 4(a)). Consequently, we developed a luciferase reporter using the wild-type as well as mutant circSYPL1 miR-506-3p genes. Cotransfection of circSYPL1-WT as well as miR-506-3p decreased luciferase activation in HEK 293T cells, but cotransfection of circSYPL1-Mut and miR-506-3p abolished the suppression of luciferase activation (Figure 4(b)). As illustrated in Figures 4(c) and 4(d), the circSYPL1 as well as miR-506-3p biotin probes were much more effective at enriching miR-506-3p and circSYPL1 by RNA pull-down assay than the negative control probe. Additionally, we determined the expression of miR-506-3p in cells administered with circSYPL1 siRNA or recombinant plasmid and discovered that circSYPL1 siRNA can significantly boost miR-506-3p levels, whereas circSYPL1 overexpression plasmids significantly lowered miR-506-3p levels (Figure 4(e)). In addition, we performed qPCR to detect miR-506-3p and found that the expression of miR-506-3p in tumor tissue was obviously decreased compared with that in adjacent tissue from patients with liver cancer (Figure 4(f)). These findings demonstrated that circSYPL1 takes part in liver cancer progression by targeting miR-506-3p.

### 3.5. miR-506-3p Targets EZH2 to Silence Its Expression

**Figure 5:**
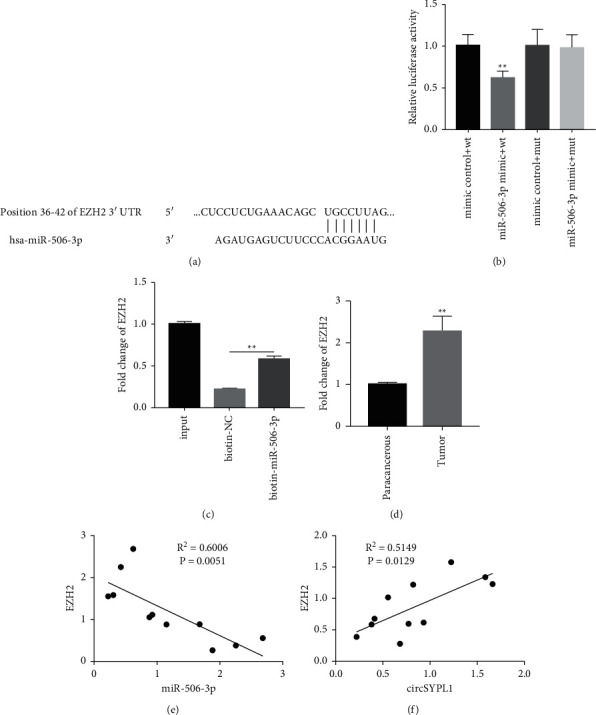
miR-506-3p targets EZH2. (a) The sequences of miR-506-3p and EZH2 are connected vertically; the seed sequence and corresponding binding sites are shown. (b) Luciferase experiments to show miR-506-direct 3p′s targeting of EZH2. (c) RNA pull-down assay to determine whether the miR-506-3p biotin probe interacts with EZH2. (d) qPCR analysis demonstrating the expression level of EZH2 in HCC and surrounding tissues. (e) Pearson's correlation analysis demonstrating that EZH2 and miR-506-3p have a negative connection. (f) Pearson's correlation analysis demonstrating that EZH2 and circSYPL1 have a positive correlation.

We carried out alignment analysis to determine the target gene of miR-506-3p. The results showed that EZH2 was a target for miR-506-3p because of the complementary interaction between EZH2 3′-UTR and miR-506-3p (Figure 5(a)). Additionally, we constructed the EZH2 WT as well as Mut 3′-UTRs into a dual-luciferase reporter system to test if miR-506-3p is effectively targeted to the EZH2 3′-UTR. We discovered that cotransfection of miR-506-3p and EZH2 3′-UTR WT dramatically decreased luciferase activation in 293T cells, especially compared to the miRNA negative control. However, the cotransfection of EZH2 3′-UTR Mut and miR-506-3p could not decrease the luciferase activity (Figure 5(b)). RNA pull-down assays showed that the miR-506-3p biotin probe could obviously enrich more EZH2 compared with the negative control probe (Figure 5(c)). Furthermore, qPCR results revealed that the expression of EZH2 in tumor tissue was obviously increased compared with adjacent tissue from patients with liver cancer (Figure 5(d)). Pearson's correlation analyses found a significant negative correlation between EZH2 and miR-506-3p levels (Figure 5(e)) and a positive correlation between circSYPL1 expression (Figure 5(f)). Such findings revealed that miR-506-3p has a role in regulating the growth of hepatocellular carcinoma by targeting EZH2.

### 3.6. miR-506-3p Overexpression Can Reverse the Upregulation of EZH2 Induced by circSYPL1

**Figure 6:**
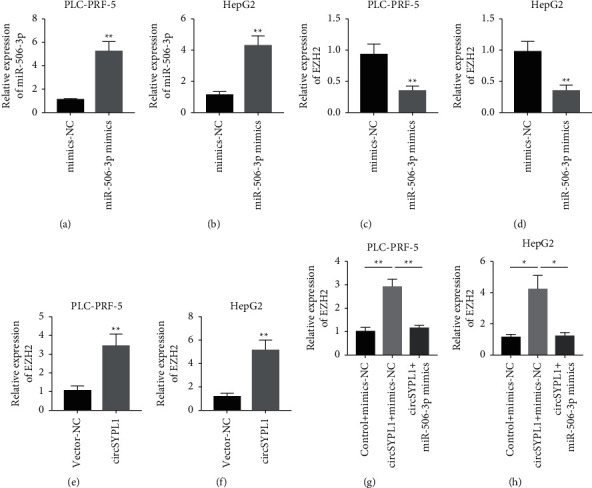
miR-506-3p overexpression could reverse the upregulation of EZH2 induced by circSYPL1. (a) qPCR showing the expression of miR-506-3p in PLC-PRF-5 cells treated with negative control mimics and miR-506-3p mimics. (b) qPCR showing the expression of miR-506-3p in HepG2 cells treated with negative control mimics and miR-506-3p mimics. (c) qPCR showing the expression of EZH2 in PLC-PRF-5 cells treated with negative control mimics and miR-506-3p mimics. (d) qPCR showing the expression of EZH2 in HepG2 cells treated with negative control mimics and miR-506-3p mimics. (e) qPCR showing the expression of EZH2 in PLC-PRF-5 cells after treating with negative control plasmid and circSYPL1 overexpressed plasmid. (f) qPCR showing the expression of EZH2 in HepG2 cells treated with a negative control plasmid or a circSYPL1 overexpressed plasmid. (g) qPCR analysis demonstrating the levels of expression of EZH2 in PLC-PRF-5 cells treated with a negative control plasmid as well as its mimics, circSYPL1 overexpressed plasmid and negative control mimics, and circSYPL1 overexpressed plasmid and miR-506-3p mimics. (h) qPCR showing the expression of EZH2 in HepG2 cells treated with negative control plasmid and negative control mimics, circSYPL1 overexpressed plasmid and negative control mimics, and circSYPL1 overexpressed plasmid and miR-506-3p mimics.

miR-506-3p as well as negative control mimics were initially synthesized to study the circSYPL1/miR-506-3p/EZH2 connection in the studied cell lines. miR-506-3p mimics were successfully transfected and suppressed the expression of EZH2 (Figures 6(a)–6(d)) as per qPCR findings. The overexpression of circSYPL1 could upregulate the expression of EZH2. The expression of EZH2 decreased after the transfection of circSYPL1 and miR-506-3p. Furthermore, we constructed circSYPL1 overexpressed plasmids and found that circSYPL1 overexpression could significantly upregulate the expression of EZH2 in the studied cell lines (Figures 5(e) and 2(f)). The expression of EZH2 in PLC-PRF-5 or HepG2 cells treated with circSYPL1 overexpressed plasmid and miR-506-3p mimic was considerably downregulated compared with that in cells treated with circSYPL1 overexpressed plasmid and negative control mimic (Figures 6(e) and 2(f)). These findings suggest that miR-506-3p overexpression could reverse the upregulation of EZH2 induced by circSYPL1.

### 3.7. circSYPL1/miR-506-3p Axis Accelerates Liver Cancer Progression via Enhancing EZH2 Expression

**Figure 7:**
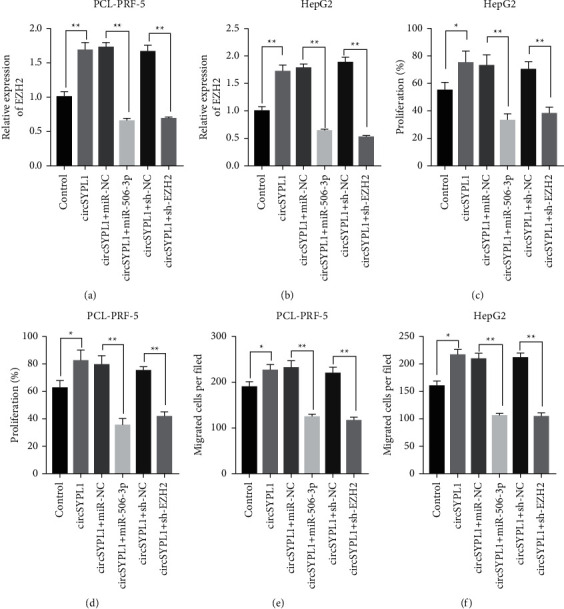
miR-506-3p overexpression or EZH2 knockdown affect cancer progression. (a) qPCR showing the expression levels of EZH2 in PLC-PRF-5 cells treated with negative control plasmid, circSYPL1 overexpressed plasmid, circSYPL1 and miR-506-3p mimics, and circSYPL1 and miR-506-3p shRNA. (b) qPCR showing the expression of EZH2 in HepG2 cells treated with negative control plasmid, circSYPL1 overexpressed plasmid, circSYPL1 and miR-506-3p mimics, and circSYPL1 and miR-506-3p shRNA. (c) PLC-PRF-5 cells treated with a negative control plasmid were analyzed for cell survival, circSYPL1 overexpressed plasmid, circSYPL1 and miR-506-3p mimics, and circSYPL1 and miR-506-3p shRNA by the CCK-8 test. (d) Cell survival assay for HepG2 cells treated with negative control plasmid, circSYPL1 overexpressed plasmid, circSYPL1 and miR-506-3p mimics, and circSYPL1 and miR-506-3p shRNA by the CCK-8 test. (e) Cell invasion assay for PLC-PRF-5 cells treated with negative control plasmid, circSYPL1 overexpressed plasmid, circSYPL1 and miR-506-3p mimics, and circSYPL1 and miR-506-3p shRNA by the transwell test. (f) Cell invasion assay for HepG2 cells treated with negative control plasmid, circSYPL1 overexpressed plasmid, circSYPL1 and miR-506-3p mimics, and circSYPL1 and miR-506-3p shRNA by the transwell test.

The overexpression or knockdown of miR-506-3p or EZH2 was tested to see if it might reverse the liver tumor development caused by circSYPL1 in order to better identify the biological role of these three molecules in the development of hepatocellular carcinoma. We have understood that circSYPL1 can increase the expression of EZH2, while miR-506-3p mimics together with circSYPL1 can significantly decrease the expression of EZH2 in liver cancer cells compared with miR-506-3p negative control and circSYPL1. In addition, EZH2 siRNA and circSYPL1 remarkably decreased the expression of EZH2 in HCC cell lines compared with EZH2 siRNA negative control and circSYPL1 (Figures 7(a) and 7(b)). CCK-8 assays showed that the cell survival capacity of HCC cell lines treated with miR-506-3p mimics and circSYPL1 was downregulated compared with that of cells treated with miR-506-3p negative control and circSYPL1. Moreover, the cell survival capacity of HCC cell lines treated with EZH2 siRNA and circSYPL1 decreased compared with that of cells treated with EZH2 siRNA negative control and circSYPL1 (Figures 7(c) and 7(d)). Furthermore, we examined the impact of the circSYPL1/miR-506-3p/EZH2 axis on cell invasion by transwell assay. HCC cell lines treated with miR-506-3p mimics and circSYPL1 had a remarkably lower number of migrating cells than those treated with miR-506-3p negative control and circSYPL1. There were also fewer migrating cells in HCC cell lines that were treated with EZH2 siRNA and upregulated circSYPL1 compared to those that were not (Figures 7(e) and 6(f)). circSYPL1 appears to help liver malignant cells grow and spread, according to these results of the study.

## 4. Discussion

circSYPL1 was discovered in this work as a novel circRNA with the potential to influence HCC development via sponging miRNAs. There was an upregulation of circSYPL1 in HCC cell lines, primary as well as metastatic tumors. This increased the aggressiveness of HCC and caused to develop in animal models. EZH2 is a downstream biomolecule which could compete with miR-506-3p to increase tumor aggressiveness. miR-506-3p overexpression or EZH2 knockdown could reverse the liver cancer progression induced by circSYPL1. Thus, circSYPL1/miR-506-3p/EZH2 could be a key signaling axis in the evolution of HCC.

A class of noncoding RNAs, known as circRNAs, has been linked to the development of HCC through its role as a ceRNA for miRNAs to influence gene stability. There are many examples of how miR-326/IGF1R enhances HCC cell proliferation [23]. When activated, CDR1as/ciRS-7 can secrete IGF-IR and certain other signaling biomolecules to impact certain signaling pathways like PI3K/AKT [24, 25]. Circ_0005986 competitively binds miR-129-5p to downregulate Notch1 mRNA, which leads to the inhibition of EMT of HCC [26]. According to current results, circRNAs appear to perform a critical function in the pathogenesis of HCC. Nevertheless, the role of circSYPL1 in the pathogenesis of HCC remains a mystery. Our research showed that circSYPL1 was upregulated in HCC cell lines and primary and metastatic tumors of patients with HCC, which could enhance the aggressiveness features of the tumor. Silencing circSYPL1 expression inhibited the malignancy of HCC, while circSYPL1 overexpression enhanced the malignancy of HCC.

miRNAs also have been extensively investigated in HCC [27]. When comparing HCC versus healthy tissues, they revealed a significant differential in the levels of miR-506, according to He and Wang [22]. A luciferase test revealed that miR-506 specifically binds to the WT 3′-UTR of YAP mRNA, but not the mutant form. HepG2 and H7402 cell growth is also considerably inhibited by miR-506. As a result of this, deleting miR-506 increases cellular proliferation. In addition to ovarian cancer [21], miR-506-3p seems to have a role in the development of other malignancies, including prostate cancer [20], OS [14, 19], nasopharyngeal carcinoma [18], pancreatic cancer [16], neuroblastoma [15], and lung cancer [28]. We actually discovered that miR-506-3p may bind to the 3′-UTR of EZH2 mRNA in our investigation and was sponged by circSYPL1, thereby upregulating EZH2 expression and enhancing the malignancy of tumors. However, miR-506-3p overexpression or EZH2 knockdown could reverse liver cancer progression induced by circSYPL1. These findings demonstrated that circSYPL1 acts as a ceRNA to sponge miR-506-3p, which enhances EZH2 expression, thereby resulting in the acceleration of HCC progression.

In our study, Pearson's correlation analysis found that the expression of EZH2 was negatively correlated with miR-506-3p and positively correlated with circSYPL1. circSYPL1 overexpression could upregulate EZH2 expression, while miR-506-3p mimics or EZH2 shRNAs reversed the circSYPL1-induced aggressiveness features of HCC cells. In breast [29] and prostate cancers [30], EZH2 has a crucial role, which is confirmed by our findings.

In this study, only subcutaneous tumor-bearing animal model was used, which cannot effectively simulate the real tumor microenvironment. Therefore, an in situ tumor model should be used in the follow-up work to further verify the role of circSYPL1. In addition, whether there are other target genes of circSYPL1 also needs to be further studied. The current conclusions should have to be translated into clinical practice through further extensive studies and research.

## 5. Conclusion

We first demonstrated that a new circRNA (circSYPL1) could competitively interact with miR-506-3p by enhancing EZH2 expression to enhance the aggressiveness characteristics of HCC cells. circSYPL1/miR-506-3p/EZH2 is an important signaling axis in HCC pathogenesis and may be a potential diagnostic biomarker and therapeutic target for HCC.

## Data Availability

The data sets analyzed or generated during the study are available from the corresponding author on reasonable request.
